# Multimodal Imaging in an Unusual Cluster of Multiple Evanescent White Dot Syndrome

**DOI:** 10.1155/2017/7535320

**Published:** 2017-05-11

**Authors:** Orly Gal-Or, Ethan Priel, Irit Rosenblatt, Shiri Shulman, Michal Kramer

**Affiliations:** ^1^Department of Ophthalmology, Rabin Medical Center-Beilinson Hospital, 4941492 Petach Tikva, Israel; ^2^MOR Institute for Medical Data, 5126413 Bnei Brak, Israel; ^3^Department of Ophthalmology, Tel Aviv Sourasky Medical Center, 62431 Tel Aviv, Israel; ^4^Sackler School of Medicine, Tel Aviv University, 6997801 Tel Aviv, Israel

## Abstract

**Objective:**

To describe an unusual cluster of multiple evanescent white dot syndrome (MEWDS) encountered within a 3-month period.

**Methods:**

This retrospective observation study is comprised of seven patients who presented with MEWDS in a 3-month period in central Israel. Data were collected from patients' medical records on clinical, multimodal imaging, and viral serology findings.

**Results:**

Six women and one man of mean age 31.5 ± 7.2 years. Three reported a precedent viral infection. All had unilateral decreased vision. Funduscopy revealed foveal granularity.

**Main Imaging Findings:**

Hyperfluorescent spots on blue autofluorescence (BAF), hypofluorescent spots on indocyanine green angiography, dark lesions on infrared photos, and ellipsoid zone irregularities on spectral domain optical coherence tomography (SD-OCT). Resolution of the spots on BAF correlated with anatomic (SD-OCT) and visual recovery. OCT angiography performed following the convalescence stage demonstrated intact retinal and choroidal flow. Serologic findings were inconclusive.

**Conclusion:**

We report a unique cluster of MEWDS patients presented in a short period of time. SD-OCT findings of ellipsoid zone disruption in combination with other multimodal imaging modalities are outlined meticulously. Recognizing these imaging features along with high index of clinical suspicion is important for the diagnosis of MEWDS. Serologic testing might be considered in future patients.

## 1. Introduction

Multiple evanescent white dot syndrome (MEWDS) was first described in 1984 as a rare, sudden onset of unilateral chorioretinopathy, with the predominant sign being multifocal yellow-white spots throughout the retina [1, 2]. The clinical spectrum of MEWDS has expanded over the years to include bilaterality and recurrences [3] or an atypical presentation involving the fovea without the white spots [4]. Symptoms include acute onset of decreased visual acuity unilaterally accompanied in most cases by photopsia and scotomata. A prodromal flu-like illness has been reported in up to 50% of cases [1]. One report described a patient with elevated levels of total serum IgG during the disease course and negative findings for IgM to herpes zoster, herpes simplex, mumps, and measles [5].

Although MEWDS is suspected to occur as a consequence of a viral-like infection in genetically susceptible individuals, its precise pathogenesis remains unknown. Recovery is gradual, over weeks to months, and the visual prognosis is very favorable [2]. Treatment is usually not required.

The incidence of MEWDS is unknown. Only small case series are reported in the literature [4–12]. One of the largest described 34 affected patients reviewed over several years' period [1, 13, 14].

The aim of the present report was to describe an unusual cluster of seven cases of MEWDS encountered within a 3-month period, with an emphasis on the clinical presentation and multimodal imaging findings. The cluster prompted us to seek a common infectious association.

## 2. Methods

A retrospective observational study was conducted in seven patients who presented with MEWDS between July and September 2013 at two tertiary medical centers in central Israel. Data on background, clinical, and laboratory parameters were collected from the medical files. The study was approved by the institutional ethics review board.

All patients underwent a comprehensive ophthalmic examination and multimodal imaging tests, including blue autofluorescence (BAF), fluorescein angiography (FA) and/or indocyanine green angiography (ICGA), infrared (IR) photography, and spectral domain optical coherence tomography (SD-OCT). Images were acquired with the HRA-2 and the Spectralis HRA + OCT devices (Heidelberg Engineering, Heidelberg, Germany) at the following wavelengths: BAF—excitation 488 nm, barrier cut-off 496 nm; IR—820 nm; ICGA—excitation 790 nm, emission 800 nm; and SD-OCT—superluminescent diode light source 870 nm. The volume scan option was used to acquire the multiple SD-OCT scans (25–49 horizontal scans over a 6 mm region covering the area of pathology). Precise registration between findings seen on IR or BAF and SD-OCT was enabled by the dual-beam laser eye-tracking system, where one laser is used to image the retina and the other laser to perform the OCT scans. Accurate rescanning in areas of interest was ensured by the Spectralis follow-up function which automatically places subsequent scans on the same location as the previous ones.

OCT angiography images were acquired using the RTVue XR Avanti with AngioVue (Optovue Inc., Fremont, California, USA), with an A-scan-rate of 70 000 scans per second, a light source of 840 nm, and a bandwidth of 45 nm. Macular cubes (3 × 3 mm) were acquired, each cube consisting of 304 clusters of 2 repeated B-scans containing 304 A-scans each. Split-spectrum amplitude decorrelation technology was employed to improve the signal-to-noise ratio by splitting the spectrum to generate multiple repeat OCT frames from 2 original repeat OCT frames [15].

Motion correction was performed using registration of 2 orthogonally captured imaging volumes. Automatic segmentation of the retinal layers was performed by the viewing software and was used to generate en face projection images after adjusting the level of the segmented layer on the B-scans.

Serology testing was performed for viruses commonly present at the time of the patients' presentation, namely, immunoglobulin IgG and IgM for herpes simplex virus (HSV) I-II, varicella zoster virus (VZV), West Nile virus, coxsackievirus, echovirus (subgroup of enterovirus), and corona virus.

## 3. Results

### 3.1. Demographics and Clinical Findings

There were one male and six female patients of mean age 31.5 ± 7.2 years (range 22–41 years).  Table 1 summarizes the demographic data. Three patients reported a prodromal virus infection.

**Figure 1:**
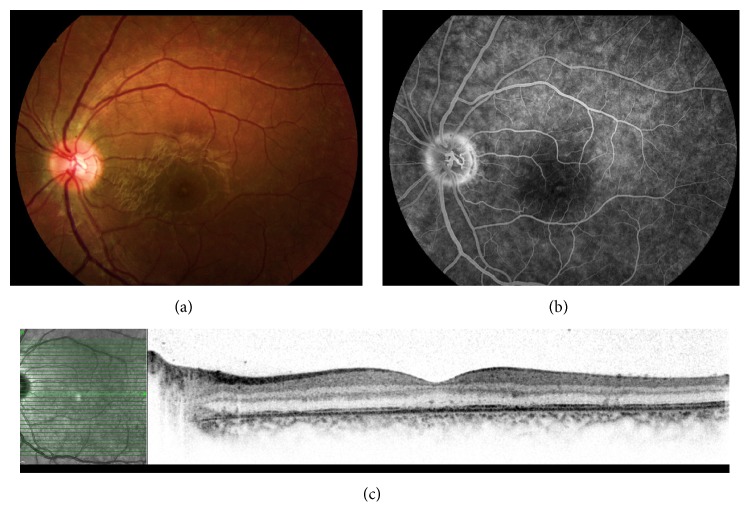
22-year-old female with visual acuity of 6/20 in her left eye (a). Color photograph of the left eye showing foveal granularity (b). FA of the left eye shows mild optic disc leakage at late phase (4 min 56 seconds) (c). SD-OCT shows disrupted ellipsoid zone more pronounced at the fovea.

**Figure 2:**
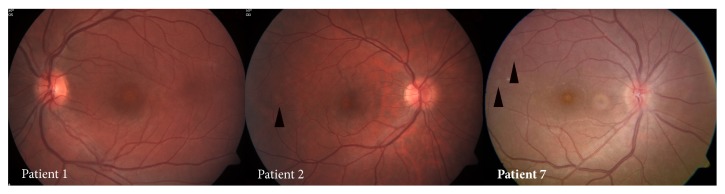
Color photograph of the left eye of patient 1 showing foveal granularity and lack of white dots (obtained 2 days after initial symptoms). Color photograph of the right eye of patient 2 showing foveal granularity with faint white retinal lesions temporal to the macula (arrowhead) (obtained 1 week after initial symptoms). Color photograph of the right eye of patient 7, demonstrating foveal granularity and scattered temporal yellow-white deep retinal lesions (arrowheads) (obtained 12 days after initial symptoms).

All patients presented with acute onset of unilateral decreased vision. The best corrected visual acuity at presentation ranged from 6/9 to 6/30 in the affected eye. None of the patients had signs of anterior or vitreous inflammation in the affected eye. Funduscopic findings at presentation included foveal granularity in six patients; in four patients (patients 1, 4, 5, and 6), it was the sole pathologic retinal finding (Figure 1); and in three patients (patients 2, 3, and 7), foveal granularity was associated with faint white retinal lesions (Figure 2), located mainly in the midperipheral retina extending to the periphery. Patient 6 had a swollen disc and mild signs of optic neuropathy (mild red desaturation, enlarged blind spot on visual field). Patient 6 underwent neurological evaluation due to initial presentation mimicking optic neuritis. Neurological evaluation including full neurological exam and neuroimaging excluded additional neurological deficit, before the diagnosis of MEWDS was established. The clinical findings are summarized in Table 2.

### 3.2. Multimodal Imaging Findings

Patients who underwent imaging less than 2 weeks from onset of symptoms had the most typical findings.

**Figure 3:**
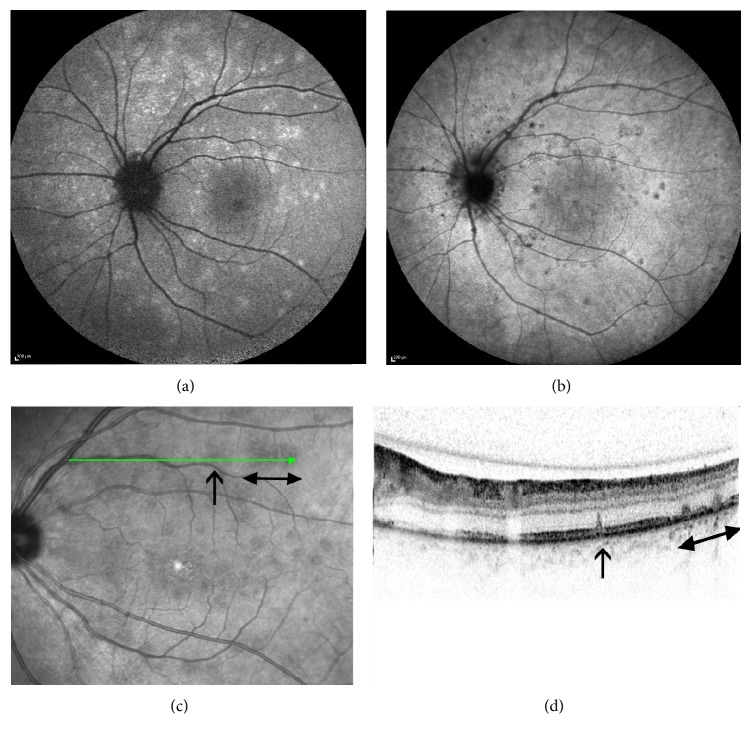
Corresponding images of patient 6: (a) BAF showing hyperautofluoresent lesions and (b) ICGA demonstrating hypofluorescent spots in similar locations, taken 1 week after initial symptom. Areas of disruption of ellipsoid zone demonstrated on SD-OCT (arrows) (d), corresponding to the dark lesions seen on IR (arrows) (c) of the same patient.

**Figure 4:**
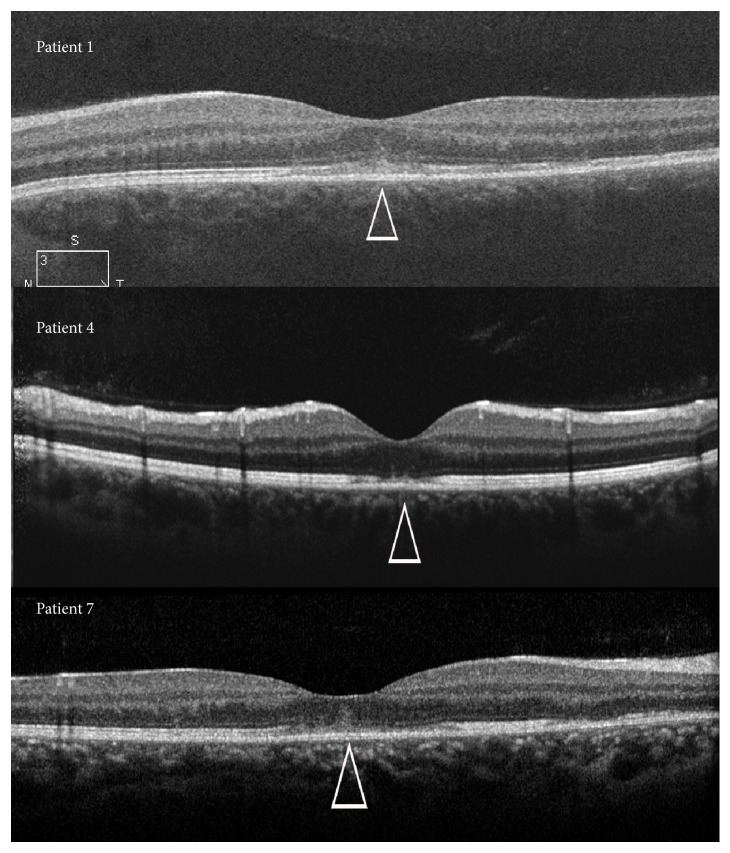
SD-OCT of 3 eyes of 3 patients, demonstrating disruption of the ellipsoid zone in the subfoveal area (arrowheads). Note the foveal hyperreflectivity extending into the inner retinal layers. Patient 1 (2 days after initial symptom). Patient 4 (1 week after initial symptom). Patient 7 (2 weeks after initial symptoms).

BAF revealed hyperautofluorescent lesions in the macula between and along the arcades in four patients (patients 1, 3, 6, and 7). IR photos showed dark lesions in similar, though not identical, locations (Figure 3). Patients 1 and 6, who underwent ICGA, had hypofluorescent lesions in numbers typically exceeding those detected by both clinical and other imaging modalities. B-scan SD-OCT through the fovea showed a disrupted inner segment ellipsoid zone band of varied severity in all 7 affected eyes. The ellipsoid zone hyper reflective band on SD-OCT anatomically correlates to photoreceptors' inner segment, ellipsoid section densely packed with mitochondria [16]. The transient disruption of the foveal ellipsoid zone on SD-OCT corresponded to the clinically apparent foveal granularity. In patient 5, who presented with sole retinal finding of foveal granularity and mild optic disc leakage on FA, the SD-OCT finding of ellipsoid zone disruption was the main sign for diagnosis MEWDS (Figure 1). Foveal hyperreflectivity found in 3 patients (patients 1, 4, and 7) was noted extending into the inner retinal layers (Figure 4). The lesions identified on the BAF, IR, and ICGA images corresponded to the areas of disruption of the ellipsoid zone, on the SD-OCT scans (Figure 3). FA demonstrated nonspecific early punctate hyperfluorescent lesions, with slight staining during the early phase, in four patients (patients 2, 3, 6, and 7). These lesions did not correspond to the findings by either the clinical or other imaging modalities. No pathology was noted in the foveal area despite the presence of typical foveal granularity. Mild optic disc leakage was evident in four patients (patients 1, 4, 5, and 6).

**Figure 5:**
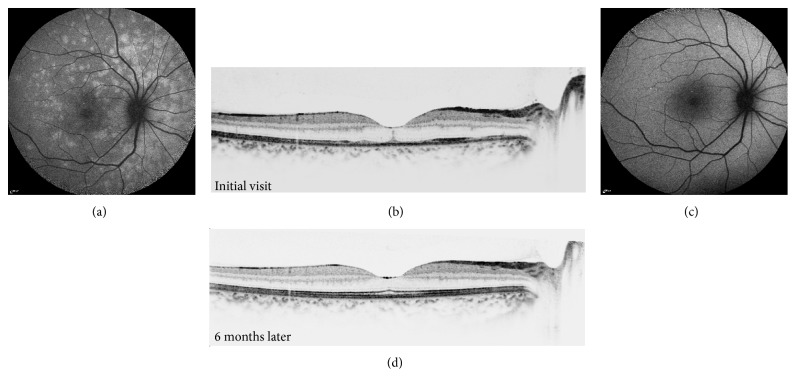
Consecutive images of patient 7. Initial visit, VA 6/18 (a, b) and at 6-month follow-up, VA 6/6.6 (c, d). BAF imaging demonstrating resolution of hyperautofluorescent spots. SD-OCT demonstrating the disruption of ellipsoid zone in the subfoveal area and restoration of the ellipsoid zone at the final visit.

During the course of the disease, the hyperautofluorescent areas decreased in number and faded without leaving hypoautofluorescent abnormalities. The resolution of the BAF lesions corresponded to the anatomic recovery observed on SD-OCT. The foveal hyperreflectivity disappeared as well (Figure 5).

**Figure 6:**
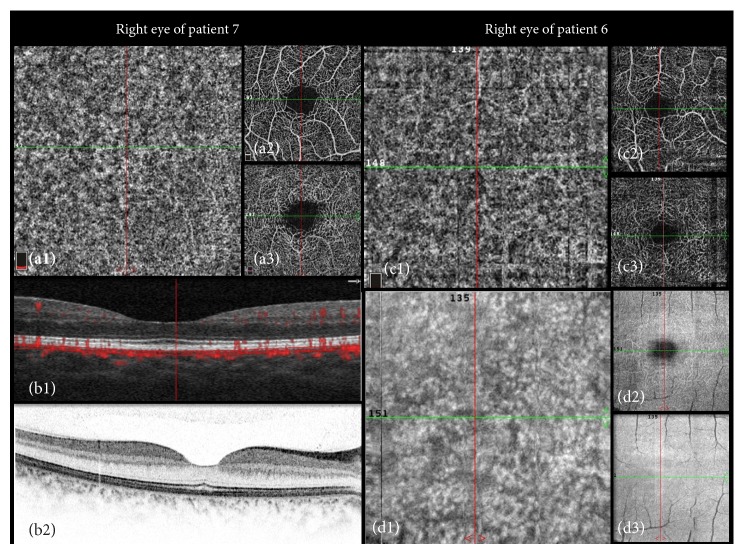
OCTA images following convalescence stage of patients 7's right eye (a-b) and 6's left eye (c-d). The green and red lines represent the *x* and *y* axes. Patient 7 after recurrent episodes. 3 × 3 mm OCT angiogram of the choriocapillaris (a1), superficial layer (a2), and deep layer (a3) centered at the macula without any flow compromise. Corresponding *x*-axis OCT structural B-scan (b1) simultaneously obtained during the same scan as the OCT angiogram with flow overlay at the cross-section demonstrated by the green line in (a1). SD-OCT (b2) demonstrating normal anatomy of the outer retina 6 months after the first acute episode. Patient 6, 3× 3 mm OCT angiogram of the choriocapillaris (c1), superficial layer (c2), and deep layer (c3) centered at the macula without any flow compromise. 3 × 3 mm en face structural OCT (d1) of the choriocapillaris centered at the macula as in c1. This image was simultaneously obtained during the same scan as the OCT angiogram in (c). En face structural OCT of the deep (d2) and outer retina (d3).

Patients 6 and 7 underwent imaging with OCT angiography at convalescence stage. Patient 7 had recurrent episodes. OCTA findings demonstrated no flow impairment in the retinal and choroidal vasculature as demonstrated in Figure 6.

Four patients (patients 1, 4, 6, and 7) underwent serological testing with negative results except for a common result of elevated titer of IgG to VZV.

After 6 months of follow-up, the best corrected visual acuity ranged from 6/6 to 6/6.6 (Table 2).

## 4. Discussion

Although MEDWS is traditionally considered as a rare syndrome [2], we report an unusual cluster of seven patients who presented within a three-month period. All patients were otherwise healthy, and all presented with decreased vision in one eye. This cluster of cases could break to some measure the statistical improbability of the rarity of the disease. The atypical presentation in most of our patients could suggest that MEWDS is underdiagnosed. However, it may be in line with the speculation that sometimes atypical findings may simply reflect the moment in time in which the patients were examined and are not a true atypical presentation [4]. In its original description by Jampol et al. [2], MEWDS cases were unilateral with fundus presentation including numerous white dots scattered in the posterior pole and beyond the arcades. During the disease course, granularity appearance of the macula develops in most cases and, when seen, determines the diagnosis. The number of white spots is very variable, and in fact, they may be absent. Given that characteristic white dots were not present in four patients (patients 1, 4, 5, and 6), we were guided by other fundus features, in particular foveal granularity, symptoms, multimodal imaging, and clinical course.

While the presumed pathogenesis of MEWDS involves a viral infection, only few reports to date have described a search for the pathogen [5, 17–19]. The present cluster of cases provided us with a unique opportunity to seek a common viral denominator. Serological testing yielded only an elevated titer of IgG to VZV, most often an indicative of past VZV infection or vaccination; thus, we could not make any generalization regarding these findings.

Multimodal imaging (BAF, SD-OCT, IR, FA, and ICGA) has proven to have high value in the challenging diagnosis of MEWDS. Most of the findings noted here have been described separately in earlier reports [7–9, 11, 12]. However, the present study offered two important advantages. We were able to examine all patients with simultaneously acquired imaging, and multiple correlations between the imaging findings and the clinical evaluation were possible. Moreover, the relatively large size of the cohort and the repeated scans allowed us to verify the imaging findings in this rare disease.

We observed corresponding locations of the dark spots on IR images, the hyperautofluorescent spots on the BAF images, and the foci of outer retinal pathology on SD-OCT images. Small hyperreflective points, located in the ganglion cell layer, the ellipsoid zone, and the choriocapillaris, have been noted and described on “en face” EDI SD-OCT [20]. However, we noted a unique finding of foveal hyperreflectivity extending into the inner retinal layers. Our finding reinforces a recently described finding in the literature [14] which is believed to be pathognomonic to MEWDS. During the disease course, both the IR and the BAF findings faded in concurrence with the anatomical resolution of the disruption in the ellipsoid zone and the foveal hyperreflective lesion on SD-OCT. Thus, IR images may provide an easy, widely available imaging modality for follow-up of patients with MEWDS.

Although IR autofluorescent changes were recently described in patients with MEWDS [21, 22], this modality is not widely available, whereas IR imaging is routinely performed. Furthermore, on the basis of our findings with multimodal imaging, we suggest that the diagnosis of MEWDS can be established with the simultaneous use of such noninvasive techniques as BAF, IR, and SD-OCT. ICGA and FA may be reserved for secondary use, when findings are equivocal. OCTA is relatively new noninvasive imaging modality that demonstrates flow characteristics of the vascular network within the regional circulation to construct noninvasive images of the vascular network. En face images generated by OCTA also allow us to study the spatial relationships between vasculature and adjacent retinal/choroidal layers with greater precision than dye angiography, and OCTA findings demonstrated no flow impairment in the retinal and choroidal vasculature of the patients scanned after convalescence stage.

We cannot overestimate the role of multimodal imaging in these patients, since not too often, the diagnosis is mistaken for optic neuritis, and clinical findings are very subtle.

Limitations of the study were the variability in time from disease onset to serologic testing, making the IgM results hard to interpret. Therefore, we consider these tests inconclusive. Secondly, not all the patients had imaging with all modalities. In addition, future research is required using OCT angiography to study the nature of the dots in MEWDS patients and its correlation to other multimodal imaging modalities in the acute and convalescent stage.

In conclusion, we present a large unique cluster of patients who presented with MEWDS over a short period of time. To the best of our knowledge, such a cluster was not previously reported in the literature nor encountered by us at different seasons. The diagnosis was supported by the presence of key features of foveal granularity and disruption of the ellipsoid zone on OCT and their correlation with the hyperautofluorescent lesions identified on BAF. Attention should also be addressed to the dark spots demonstrated on IR images, which may serve as an additional diagnostic clue provided by a noninvasive imaging modality. The disease course in our patients was typical for MEWDS, with almost complete recovery of visual acuity. The specific pathogenesis of MEWDS is unknown but is believed to be an inflammatory condition following a viral infection. We suggest continued serological testing in patients who meet the clinical criteria. The clinical signs of MEWDS are subtle, such that the diagnosis relies on a high index of suspicion.

## Figures and Tables

**Table 1:** Demographic and clinical data.

Patient number	Sex	Age (yr)	Viral prodrome	Time of presentation
1	F	30	−	July 2013
2	M	32	−	July 2013
3	F	39	−	July 2013
4	F	34	+	July 2013
5	F	22	−	July 2013
6	F	41	+	August 2013
7	F	23	+	September 2013

+: present; −: absent.

**Table 2:** Clinical and multimodal imaging findings.

Pt. number	VA at presentation	VA at last follow-up	Clinical examination			BAF lesions	IR lesions	SD-OCT EZ disruption	FA		ICG	OCTA
			Foveal granularity	Swollen disc	Spots/dots				Optic disc leakage	Spots		Flow compromise of choriocapillaris and retinal vasculture
1	6/12	6/6	+	−	−	+	+	+	+	−	+	
2^∗^	6/18	6/6.6	+	−	+		+	+	−	+		
3	6/9	6/6	−	−	+	+	−	+	−	+		
4^∗^	6/30	6/6	+	−	−		+	+	+	−		
5	6/20	6/6.6	+	−	−	−	−	+	+	−		
6^∗∗^	6/10	6/6.6	+	+	−	+	+	+	+	+	+	−
7^∗∗^	6/18	6/6.6	+	−	+	+	+	+	−	+		−

^∗^In patients 2 and 4, BAF was not done; ^∗∗^OCTA was performed following convalescence stage; VA: visual acuity of affected eye; BAF: blue autofluorescence; IR: infrared; FA: fluorescein angiography; SD-OCT: spectral domain optical coherence tomography; ICG: indocyanine green; OCTA: optical coherence tomography angiography; EZ: ellipsoid zone.
